# Characterisation of patients with familial chylomicronaemia syndrome (FCS) and multifactorial chylomicronaemia syndrome (MCS): Establishment of an FCS clinical diagnostic score

**DOI:** 10.1016/j.dib.2018.10.125

**Published:** 2018-10-27

**Authors:** Philippe Moulin, Robert Dufour, Maurizio Averna, Marcello Arca, Angelo B. Cefalù, Davide Noto, Laura D’Erasmo, Alessia Di Costanzo, Christophe Marçais, Luis Antonio Alvarez-Sala Walther, Maciej Banach, Jan Borén, Robert Cramb, Ioanna Gouni-Berthold, Elizabeth Hughes, Colin Johnson, Xavier Pintó, Željko Reiner, Jeanine Roeters van Lennep, Handrean Soran, Claudia Stefanutti, Erik Stroes, Eric Bruckert

**Affiliations:** a Hôpital Cardiovasculaire Louis Pradel, Hospices Civils de Lyon, INSERM UMR 1060 Carmen, Université Claude Bernard Lyon 1, Lyon, France; b Institut de Recherches Cliniques de Montréal, Montréal, Canada; c Dipartimento Biomedico di Medicina Interna e Specialistica (Di.Bi.M.I.S.), University of Palermo, Palermo, Italy; d Department of Internal Medicine and Medical Specialties, Sapienza University of Rome, Rome, Italy; e Hospital General Universitario Gregorio Marañón, IiSGM, Department of Medicine, School of Medicine, Universidad Complutense de Madrid, Madrid, Spain; f Medical University of Lodz, Lodz, Poland; g University of Gothenburg, Gothenburg, Sweden; h University Hospitals Birmingham NHS Foundation Trust, Birmingham, UK; i University of Cologne, Cologne, Germany; j Sandwell and West Birmingham Hospitals NHS Trust, Birmingham, UK; k University Hospital, Southampton, UK; l Bellvitge University Hospital, Barcelona, Spain; m University Hospital Center Zagreb, Zagreb, Croatia; n Erasmus Medical Centre, Rotterdam, the Netherlands; o Central Manchester University Hospital NHS Foundation Trust, Manchester, UK; p Department of Molecular Medicine, Sapienza University of Rome, Rome, Italy; q Academic Medical Center, Amsterdam, the Netherlands; r Endocrinologie Métabolisme et Prévention Cardiovasculaire, Institut E3M et IHU Cardiométabolique (ICAN), Hôpital Pitié Salpêtrière, 47–83 Boulevard de l’Hôpital, 75013 Paris, France

## Abstract

Data presented in this article are supplementary material to our article entitled “Identification and diagnosis of patients with familial chylomicronaemia syndrome (FCS): expert panel recommendations and proposal of an “FCS Score” (Moulin et al., 2018, in press). The data describe the genotypes of patients with familial chylomicronaemia syndrome (FCS) and multifactorial chylomicronaemia syndrome (MCS), from the validation and replication cohorts.

**Specifications table**

**Table:** 

Subject area	*Medicine*
More specific subject area	*Hypertriglyceridaemia*
Type of data	*Text file, Table*
How data was acquired	*Retrospectively. Clinical history and genotyping of patients*
Data format	*Summary of raw data*
Experimental factors	*Retrospective analysis of patient records*
Experimental features	*The cut-off for the familial chylomicronaemia syndrome score was determined from a validation cohort and tested on replication cohorts*
Data source location	*Lyon, France; Montréal, Canada; Rome, Italy; Palermo, Italy*
Data accessibility	*Data are within this article*

**Value of the data**

• Summary data from relatively large cohorts of familial chylomicronaemia syndrome (FCS) and multifactorial chylomicronaemia syndrome (MCS) patients.
• The data illustrate how a cut-off level of ≥10 for the FCS clinical diagnostic score [1] may help to differentiate between FCS and MCS patients.
• The data provide a benchmark for future studies.

## 1 Data

The familial chylomicronaemia syndrome (FCS) cohort included 25 patients with FCS from the Montreal lipid clinic and four patients from the Lyon lipid clinic (Table 1). The multifactorial chylomicronaemia syndrome (MCS) cohort included 29 patients consecutively studied over the previous 2 years in the Lyon lipid clinic (Table 1). The FCS cohort was used to establish sensitivity and the MCS cohort was used to establish specificity, leading to a receiver operating characteristic (ROC) curve area of 0.91 [1]. Replication of the diagnosis capacity of the FCS score was retrospectively tested in two additional lipid clinics. The Rome replication cohort included 16 patients with FCS and 15 patients with MCS (Table 1). The Palermo replication cohort included eight patients with FCS and eight patients with MCS (Table 1).

**Table 1:** Hypertriglyceridaemic patients: genotypes found in the different cohorts.

	**FCS**					**MCS**			
	**Ho LPL**	**Comp He LPL**	**Ho not LPL**	**Comp He not LPL**	**WT low LPL activity**	**He**	**Pol**	**WT**	**NA**
Montreal	15	7	1	0	2				
Lyon			3	1		11	8	5	5
Rome	8	1	5	2		11		4	
Palermo	6	0	2			1	2	3	2

FCS, familial chylomicronaemia syndrome; MCS, multifactorial chylomicronaemia syndrome; Ho, homozygous; LPL, lipoprotein lipase; Comp, compound; He, heterozygous; WT, wild type; Pol, multiple functional SNPs; NA, not available.

## 2 Experimental design, materials and methods

The items of the FCS score were selected on a pragmatic basis following discussion within a panel of experts. The relative weight of each item was set up also on a pragmatic basis. The cut-off was determined from a validation cohort and tested on replication cohorts. FCS patients were defined as any patient carrier of a homozygous or a compound heterozygous loss of function mutation in lipoprotein lipase (*LPL*), apolipoprotein C2 (*APOC2*), apolipoprotein A5 (*APOA5*), glycosylphosphatidylinositol-anchored high-density lipoprotein-binding protein 1 (*GPIHBP1*) and lipase maturation factor 1 (*LMF1*) genes or a low post-heparin LPL activity. MCS patients were defined as patients with documented history of plasma triglyceride (TG) >10 mmol/L and carriers of either a heterozygous loss of function mutation and/or variants associated with increased TG level in *LPL, APOC2, APOA5, GPIHBP1* and *LMF1* genes.

In the patients with MCS, due to the retrospective design, the plasma TG concentration was considered to be consistently >10 mmol/L in order to challenge the specificity of the FCS score, if not enough information was available in the medical file regarding the reproducibility of the plasma TG concentration >10 mmol/L. Further study is needed to prospectively validate the score in cohorts with comprehensive phenotype available.

All the patients gave written, informed consent for genotyping. All the French patients received written information regarding the study according to the French bioethics Law Jardé 2017.
