# Supplementary material for: Characterisation of patients with familial chylomicronaemia syndrome (FCS) and multifactorial chylomicronaemia syndrome (MCS): Establishment of an FCS clinical diagnostic score
Source: Data Brief. 2018 Oct 27;21:1334–6. doi: 10.1016/j.dib.2018.10.125 (PMC6231039; doi:10.1016/j.dib.2018.10.125)
Supplement: Supplementary file 1 — Supplementary material [file mmc1.docx]

**Conflict of interest**

PM has received fees paid to his institution for membership of advisory boards and

clinical trials for Akcea/Ionis, Aegerion, Amgen, AMT/Chiesi, MSD, Novartis,

Regeneron and Sanofi. RD has received honoraria for consulting from Amgen,

Sanofi/Regeneron and Akcea/Ionis. MAv has received honoraria for membership of

advisory boards and Speaker Bureaus for Akcea/Ionis, Aegerion, Amgen, MSD,

Regeneron and Sanofi. MAr has received honoraria for membership of advisory

boards and Speaker Bureaus for Akcea/Ionis, Aegerion, Amgen, Regeneron and

Sanofi. ABC has received honoraria for Speaker Bureaus for Alpha-Sigma, Aegerion,

MSD and Sanofi. DN has received honoraria for lectures from MSD and Sanofi.

LD’E has received grants from Aegerion/Amryt. ADC has nothing to declare. CM

has nothing to declare. LAA-SW has received honoraria for Speaker Bureaus and

lectures for Amgen, Regeneron/Sanofi, Sanofi-Aventis, MSD, Pfizer, Bayer, Ferrer,

Esteve and Rovi, and consultancy fees for Akcea, MSD, Pfizer and Rovi. MB has

received honoraria for Speaker Bureaus for Abbott/Mylan, Abbott Vascular, Actavis,

Akcea, Amgen, Biofarm, KRKA, MSD, Sanofi-Aventis, Valeant and Zentiva,

consultancy fees for Abbott Vascular, Akcea, Amgen, Daichii Sankyo, Esperion,

Lilly, MSD, Resverlogix and Sanofi-Aventis, and grants from Sanofi and Valeant. JB

has received research grants from Amgen, AstraZeneca, NovoNordisk, Pfizer and

Regeneron/Sanofi, and honoraria for consultancy and lectures from Amgen,

AstraZeneca, Eli Lilly, Merck, NovoNordisk, Pfizer and Regeneron/Sanofi. RC has

presented data and chaired a meeting for Akcea, been on an advisory panel for

Novartis, and is a trustee for HEART UK, The Cholesterol Charity. IG-B has

received honoraria for consulting from Amgen, Sanofi, Eli Lilly, Regeneron, Akcea

and Aegereon. EH reports personal fees from Akcea, outside the submitted work. CJ

has received honoraria for consulting from Mylan, Abbott, UniQure and Akcea. XP

has been involved in advisory boards with Amgen, Sanofi, Rubió, Ferrer and Esteve,

has received honoraria from Akcea, and has given lectures for Mylan and Rubió. ZR

has received honoraria for consulting from Akcea. JRvL has received honoraria from

Akcea and grants from Aegerion/Amryt. HS reports personal fees from Akcea

Therapeutics UK Ltd, during the conduct of the study, and grants and personal fees

from Akcea Therapeutics UK Ltd, Amgen, Sanofi, Alexion Pharmaceuticals and

Chiesi, outside the submitted work. CS has nothing to disclose. ES reports lecturing

fees paid to his institute by Amgen, Sanofi, Chiesi, AstraZeneca, Regeneron and

Akcea. EB declares having received honoraria for consulting/presentation from

AstraZeneca, Amgen, Genfit, MSD, Sanofi, Regeneron, Unilever, Danone, Aegerion,

Chiesi, Rottapharm-MEDA, Lilly, Ionis Pharmaceuticals and Akcea.
